# Carrion use by a reptile is influenced by season, habitat and competition with an apex mammalian scavenger

**DOI:** 10.1002/ece3.70211

**Published:** 2024-08-28

**Authors:** Rhys J. Cairncross, Emma E. Spencer, Niraj Meisuria, Mathew S. Crowther, Thomas M. Newsome

**Affiliations:** ^1^ School of Life and Environmental Science The University of Sydney Sydney New South Wales Australia

**Keywords:** *Canis dingo*, carrion, reptile, resource hotspot, scavenging, *Varanus varius*, *Vulpes vulpes*

## Abstract

Scavenging on carrion is critical and often fiercely competitive for a range of vertebrate species, from native apex predators to invasive species and even reptiles. Within Australia, a notable reptilian scavenger is the lace monitor (*Varanus varius*). In this study, we quantified lace monitor activity at carcasses and compared their use of the resource to common co‐occurring predators that also scavenge; the invasive red fox (*Vulpes vulpes*) and a native apex predator, the dingo (*Canis dingo*). To do so, we deployed 80 macropod carcasses equally across seasons (summer and winter) and habitats (open and closed canopy) in a temperate bioregion and monitored vertebrate scavenging with camera traps. Lace monitor activity (visitation at carcass sites inclusive of both non‐scavenging and scavenging events) was 1.67 times higher in summer than in winter, but it did not differ across closed and open habitats. Monitor activity occurred earlier after carcass deployment at sites deployed in summer than winter (1.47‐fold earlier), and at carcasses in open than closed habitats (0.22‐fold earlier). Lace monitors initially discovered carcass sites faster in summer than winter and before both red foxes and dingoes in summer. The species was active diurnally in both summer and winter, differing from the red fox, which was strictly a nocturnal scavenger and the dingo, which was significantly more active at night across both seasons. Finally, we found that lace monitor activity at carcass sites decreased slightly with higher rates of activity for dingoes (0.04‐fold decrease as dingo activity increased), but not with red fox activity. Our results have implications for understanding lace monitor foraging and scavenging and highlight the value of monitoring carcasses to provide important insights into the behaviour of varanid lizards that scavenge.

## Graphical abstract

We assessed how the activity of a reptile scavenger at carrion, the lace monitor, was influenced by biotic and abiotic variables. Lace monitors were more active at carcasses in summer than in winter but were not influenced by habitat variables. Lace monitors were faster to discover carrion in summer than dingoes and red foxes but were negatively correlated with high rates of dingo activity.

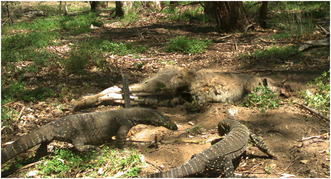

## 1 INTRODUCTION

Carrion (dead animal matter) is an important resource that contributes towards biodiversity and a range of ecological processes (Barton et al., 2013). It is a focal food source for biota that scavenge, both opportunistically, as facultative scavengers, and by necessity, as obligate scavengers. Animal carcasses are deposited in ecosystems due to a variety of reasons, from biological causes like disease and predation (Moleón et al., 2019), to die‐offs triggered by drought and heat waves, as well as anthropogenic culling programmes, hunting and vehicular collisions (Alexander, 1997; Letnic & Crowther, 2013; Pedler et al., 2021; Saunders & Doley, 2019; Watter et al., 2020; Wilmers et al., 2003). These carcasses are generally produced in a spatially and temporally patchy manner across the environment, but typically have high nutritional value (Barton et al., 2013). For this reason, competition for carcass resources can be particularly fierce (Allen et al., 2021).

Vertebrate scavenging may be shaped by competition from insects and decomposers like microbes, especially during warmer seasons (DeVault et al., 2004). However, vertebrate scavengers will also compete with, and even attack and kill, each other to monopolise carrion resources. For example, apex scavengers, large‐bodied scavengers that are often apex predators, may yield predation or fear effects to kill or deter smaller scavengers, and they are often also capable of consuming large amounts of carcasses to outcompete subordinate taxa (Tobajas et al., 2021). Despite this, smaller scavengers, or mesoscavengers, can be highly active at carrion and may quickly become abundant in the absence of apex scavengers (Forsyth et al., 2014; Hill et al., 2018; O'Bryan et al., 2019). Many mesoscavengers will avoid carrion when apex scavengers are present at or near the resource, instead changing their activity times to reduce confrontations (Allen et al., 2024; Olea et al., 2022; O'Malley et al., 2018). For instance, within scavenger communities in North America, coyotes (*Canis latrans*) and red foxes (*Vulpes vulpes*) may alter their activity patterns to avoid interactions with larger animals like wolves (*Canis lupus*), bears (*Ursus* sp.) and mountain lions (*Puma concolor*) (Allen et al., 2014, 2021, 2024; O'Malley et al., 2018; Wilmers et al., 2003). Similarly, in Australia, smaller (invasive) scavengers like the red fox may avoid larger mammalian scavengers like the dingo (*Canis dingo*) at carrion sites (Forsyth et al., 2014).

**FIGURE 1:**
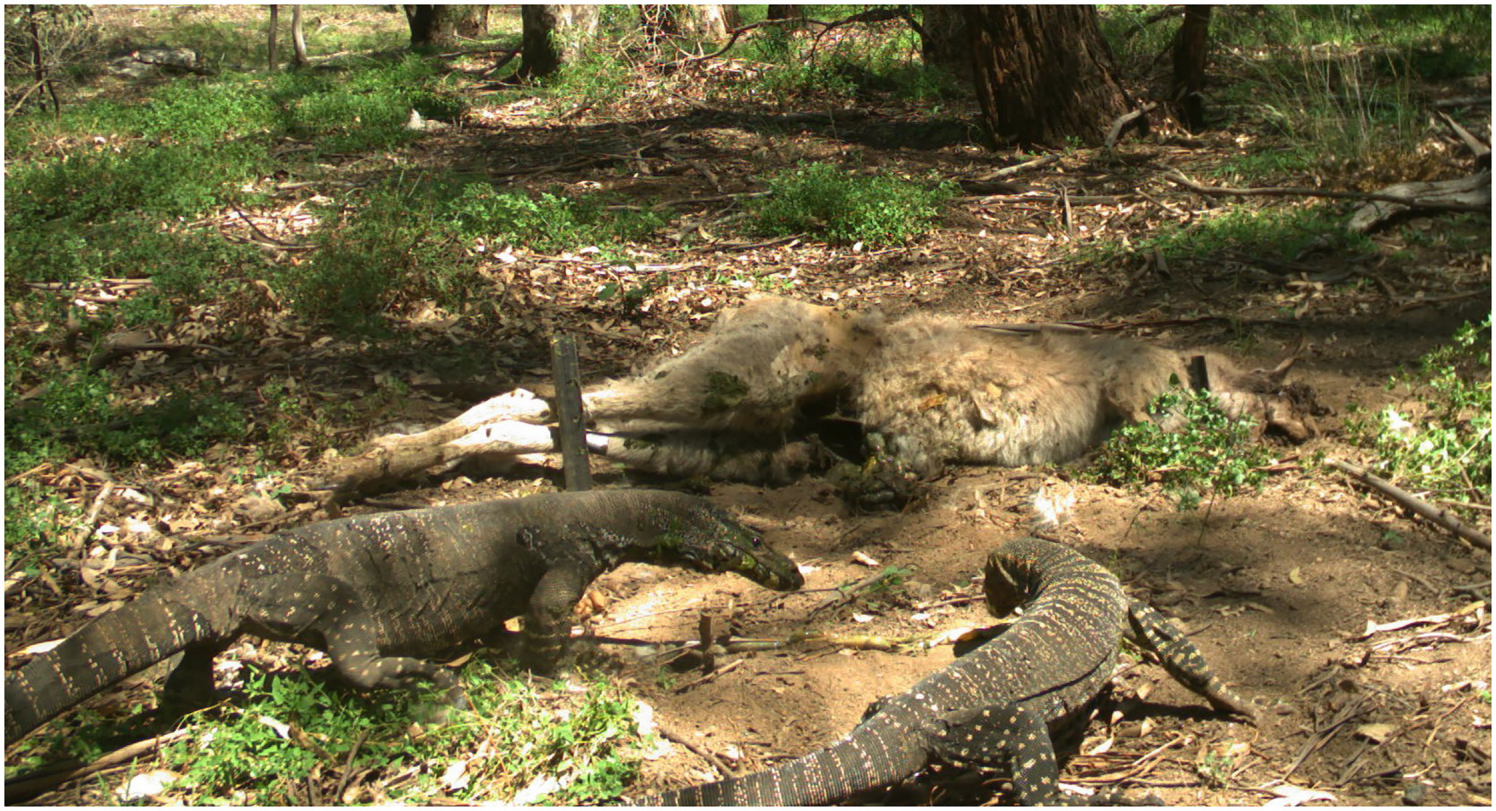
Two adult lace monitors (*Varanus varius*) at a kangaroo carcass site.

Uniquely, Australia is also home to a diversity of terrestrial lizards from the family Varanidae (Cogger, 2014), most of which are facultative mesoscavengers (Bolton & Moseby, 2004; Guarino, 2001; Jameson et al., 2024; King et al., 1989; O'Brien et al., 2007; Pettit, Ward‐Fear, & Shine, 2021; Weavers, 2014). Yet, the drivers and dynamics of reptile activity at carrion, particularly of varanid lizard species, has not been well quantified generally. One such species is the lace monitor (*Varanus varius*) (Figure 1), which is the dominant terrestrial reptilian predator across much of eastern Australia (Smissen et al., 2013). This species has a generalist diet that is highly plastic, allowing it to shift foraging behaviours dependent upon the availability of food sources (Jessop et al., 2010, 2012), enabling it to capitalise on carrion where it occurs (Losos & Greene, 1988).

Lace monitor foraging behaviours and activity may be influenced by a variety of factors. For instance, higher temperatures in warmer seasons generally facilitate increased lace monitor activity, owing to their ectothermic physiology (Guarino, 2002; Guarino et al., 2002; Stebbins & Barwick, 1968), although these species will move and forage in winter (cooler months June, July and August) on warmer, sunny days (Pascoe et al., 2019). The ability for lace monitors to remain active, at varying levels, across the year means they will often find carrion, since this resource is available in higher quantities across seasons in Australia, for instance after management culls of overabundant herbivores (Guarino, 2001). Lace monitors also exhibit a preference for occupying wooded areas (Jessop et al., 2022; Lei & Booth, 2018a, 2018b), largely because tree‐hollows provide refugia important for thermoregulation and shelter (Cotsell & Vernes, 2016; Guarino et al., 2002; Pascoe et al., 2019; Stebbins & Barwick, 1968). Lace monitors are, however, capable of also surviving in partly disturbed landscapes that have experienced some land‐clearing but where sufficient wooded patches remain, such as agricultural areas, although this is not habitat they favour (Jessop et al., 2012; Kay et al., 2018; Stebbins & Barwick, 1968). Nonetheless, they are not wholly dependent on trees for shelter, capable of using burrows and terrestrial shelters like other varanids (Thompson, 1992; Weavers, 1993). In such landscapes, they forage on a variety of food sources that may differ from their natural dietary niches (Pettit, Brown, et al., 2021), including large ungulate carcasses (Pascoe et al., 2011), adaptively utilising whatever resources are available.

Competition and predation from other species also drives lace monitor foraging patterns and may dictate their behaviours and physiology (Hu et al., 2019; Jessop et al., 2015). For instance, lace monitors have higher levels of stress hormones and lower bodily conditions in regions with unsuppressed red fox populations (Anson et al., 2013; Jessop et al., 2015) and higher abundance in areas where red foxes are controlled (Jessop & Gillespie, 2022). Whilst red foxes may not regularly prey on adult lace monitors, they feed on juveniles and eggs (Stobo‐Wilson et al., 2021), with squamates, including other, smaller, species of varanid lizard known to be prominent items in the diet of red foxes (Olsson et al., 2005; Stobo‐Wilson et al., 2020, 2021). More pertinently, when sympatric, these two species are in direct competition for prey, such that greater red fox activity may exert negative effects on lace monitors via exploitation competition of similar resources, possibly including carrion (Anson et al., 2014; Hu et al., 2019). Similarly, dingoes may affect lace monitors and their behaviour. Dingoes are capable of killing large adult lace monitors (Webb, 1996) and a continent‐wide analysis of dingo diets reveal varanids, among other reptiles, as food sources (Behrendorff et al., 2016; Doherty et al., 2019). Dingoes are also capable of removing significant amounts of carcass biomass, precluding other species from feeding on it at a single carcass as well as across landscapes when dingo activity at carcasses is high more broadly (Spencer & Newsome, 2021), potentially including the lace monitor. In combination, lace monitors may alter their behaviour and activity around carrion in response to use of the same resource by red foxes and dingoes.

Here we assess lace monitor use of carrion across two distinct habitats (open grasslands and closed woodland/open forest) and seasons (cool winter and warm summer) within a temperate bioregion of southeast Australia. We also compare the activity of lace monitors at carrion to dingoes and red foxes, exploring temporal use of carcasses by all three species. We predicted that: (1) lace monitor activity at carrion would be higher in summer and closed canopy habitats. We also predict that lace monitor events at carcasses would occur earlier in decomposition (regardless of season and habitat) when there is more, fresher, carcass biomass, because scavengers typically favour foraging at carrion when there is more of the resource available (Bragato et al., 2022); (2) dingoes, as an apex scavenger, would locate carcasses faster than lace monitors, which would find carrion at similar times to red foxes, another mesoscavenger; (3) there would be temporal (within day) separation in the use of carrion by lace monitors compared to dingoes and red foxes, in part because lace monitors are diurnal whilst dingoes and red foxes often favour scavenging at dusk or night (Forsyth et al., 2014), but potentially also in response to competition via influence of predation/fear effects and (4) carcass sites with higher dingo and red fox activity would have lower lace monitor activity, in part due to competition with both species, via influence of predation/fear effects, but also the ability of dingoes and red foxes to remove carcass biomass and thus make the carcass site less attractive for scavenging (see Appendix S1 for more information).

## 2 METHODS

### 2.1 Study area and experimental design

**FIGURE 2:**
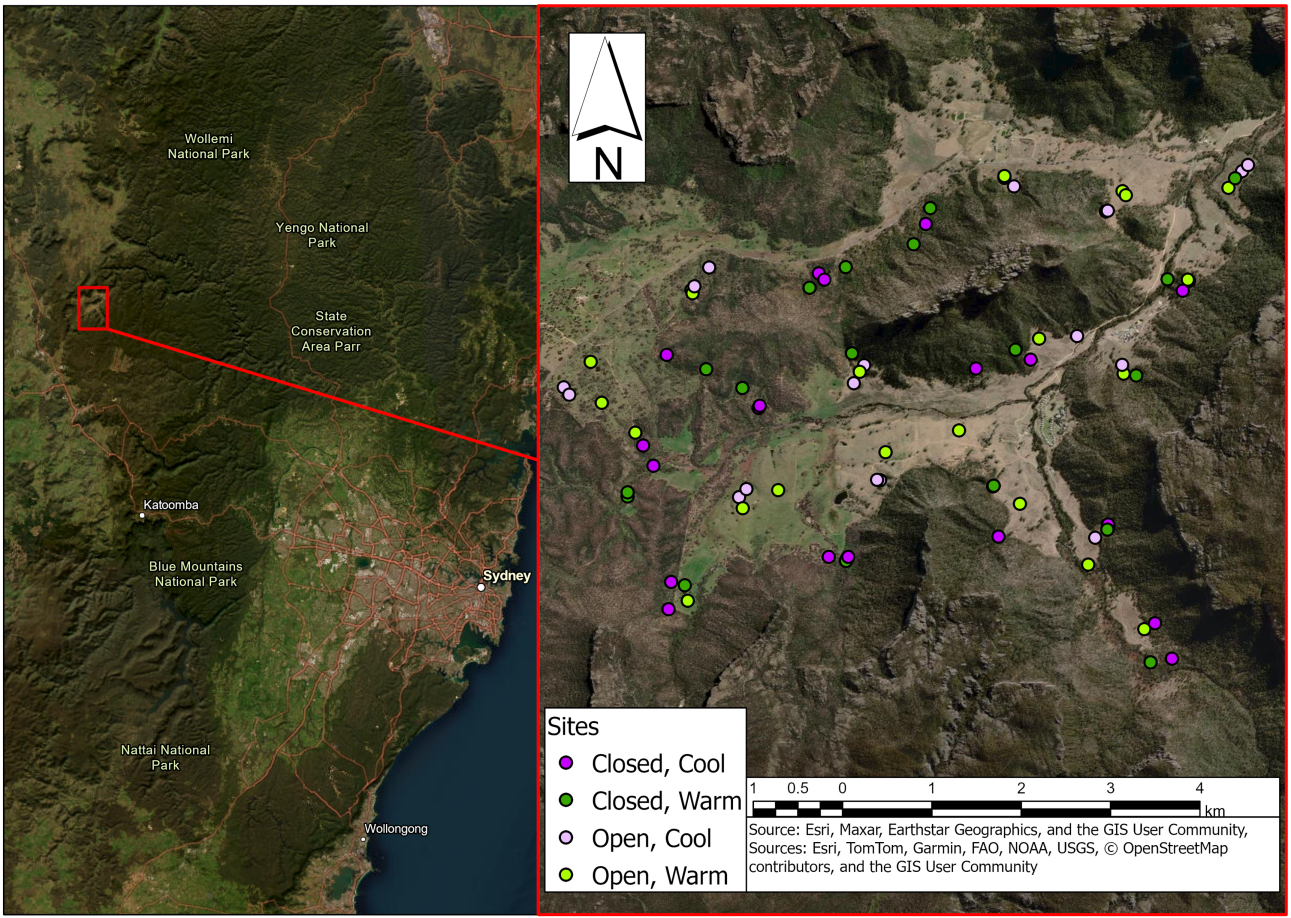
Map of the study area showing location of the 80 carcass sites monitored across the four periods of study (*n* = 2 winter, *n* = 2 summer). Site locations were interspersed and randomly shifted 200 m with each carcass deployment to minimise any potential spatial autocorrelation and confounding.

The study was undertaken within the Wolgan Valley in the western Blue Mountains, southeast Australia (Figure 2). This study area consists of a matrix of grassland (open habitat) and woodland/open forest (closed habitat) systems. Open habitats consisted of derived native grassland with the proliferation of exotic species introduced by agricultural activities. The predominant native grass was *Rytidosperma caespitosum* and *Microlaena stipoides*, whilst exotic species present included *Andropogon virginicus*. Closed habitats consisted of a heterogenous mix of canopy trees, particularly *Eucalyptus haemastoma*, *Eucalyptus punctata* and *Eucalyptus macrorhyncha* supporting a mostly native mid‐ and understorey. The study area occurs within the Sydney Basin bioregion and is climatically temperate, experiencing warm summers and mild to cool winters with no dry season (NSW NPWS, 2003). Mean maximum temperatures range between 22.4 and 31.9°C and mean minimum temperatures range between −1.4 and 8.1°C. Rainfall varies annually depending upon a range of factors including the Indian Ocean dipole and El Nino Southern Oscillation (Pepler et al., 2014) but mean annual rainfall is typically 522–2395 mm (NSW NPWS, 2003).

To track lace monitor and co‐occurring scavenger activity at carrion we deployed eastern grey kangaroo (*Macropus giganteus*) carcasses at 20 sites interspersed evenly between open and closed habitats (*n* = 10 in open and *n* = 10 in closed habitat areas) throughout the study area (Figure 2). Kangaroo carcasses are an appropriate carrion source since high numbers are culled annually in Australia as part of population management or die‐off during unfavourable conditions, including within landscapes such as the study area where lace monitors are abundant (Alexander, 1997; Letnic & Crowther, 2013; Pedler et al., 2021). This was repeated across four independent carcass deployment periods, with two carcass deployments occurring in winter and two in summer months (Figure 2), such that a total of 80 kangaroo carcasses were monitored over the four periods. Carcass monitoring occurred during August 2017 and July 2019 in winter and January 2018 and 2019 in summer. Within each monitoring period, carcass sites were positioned at least 1 km from the next closest site to mitigate the potential for confounded scent cues (Perez et al., 2016). This is considered an appropriate distance for the scent detection ranges of red foxes, dingoes and lace monitors and has been applied in other studies (Jameson et al., 2024; Leo et al., 2015; Walker et al., 2024). Between monitoring periods, carcass sites were moved at least 200 m from their position during the prior deployment period, to reduce the chance that scavenging animals became habituated to the food source. Carcasses had an average weight of 30.1 ± 9.6 (SD) kg and were sourced from external management culls under Scientific Licence SL101901 and University of Sydney Animal Ethics Committee Approval 2017/1173.

To monitor the carcasses, we placed a single camera trap, Reconyx PC800 Hyperfire™ (Professional Reconyx Inc., Holmen, WI, USA), on a star picket approximately 3–4 m from the carcass. Carcasses were placed into the field within 24 h of death and were never frozen. Each was fastened to two metal stakes hammered into the ground with the legs always facing the camera. The neck was secured to one stake and the hind legs to the other. Cameras were programmed to take 10 continuous photographs when a thermal signature and/or motion was detected with no‐delay (rapidfire) between photo bursts. As reptiles may be more difficult to detect on camera traps than other species (Hobbs & Brehme, 2017), we ensured all cameras were deployed with the highest sensitivity settings, as per other studies that have monitored varanids via camera (Aiyer et al., 2022; Jameson et al., 2024). We also undertook a ‘walk test’ to ensure cameras were framed to capture an adequate field‐of‐view around the carcass, with it centred in the middle of photographs. In all analyses, we included data from the first 30 days of monitoring after carcass deployment, thereby allowing us to capture the main period that vertebrate scavenging occurs (Bragato et al., 2022; Spencer & Newsome, 2021).

### 2.2 Statistical analysis

Camera trap photographs were processed and species identified in the program ‘DigiKam’ (Version 8.3.0). We created tags identifying all species, which we then filtered to focus on our target species: lace monitors, red foxes and dingoes. We focused on these species since they were the only larger terrestrial predators recorded that were likely to directly influence lace monitors. Feral cats (*Felis catus*), which may feed on carrion and smaller lace monitors (Woinarski et al., 2018), were recorded in very low abundance in this study. Avian scavengers like wedge‐tailed eagles (*Aquila audax*) were also not included in our analysis. Wedge‐tailed eagles are often present at carrion and can remove carcass biomass efficiently (Peisley et al., 2017) but were recorded in low abundance during monitoring. Furthermore, whilst possibly able to prey on small, younger, lace monitors, this species generally prefers mammals and previously recorded reptilian prey has typically been smaller varanids, such as the sand goanna (*Varanus gouldii*) (Aumann, 2001; Brooker & Ridpath, 1980). Other avian scavengers like corvids are common at carrion but are unable to outcompete lace monitors since they do not remove large amounts of carcasses quickly and do not prey on varanids. All data were extracted from the tagged images using the package ‘camtrapR’ (Niedballa et al., 2016). For analyses, this data was grouped separately into independent ‘events’ for lace monitors, red foxes and dingoes. An event was defined as the sequence of photographs taken from when an individual or group of the respective species was first recorded in a photograph, to when the species was last seen in that sequence of images. A time gap of less than 10 min between photographs of the same species was considered to be within the same event. Any images taken beyond 10 min of the previous photograph were considered part of a new event. To understand the activity of these animals at carcasses, we included all visitation events in the analysis, regardless of if a species scavenged the carcasses or not. We did this since the presence of an animal at a carcass, even if only brief, may influence other animals, such as via the production of scent markers that may attract or repel animals accordingly (O'Malley et al., 2018).

To test part of prediction 1 (i.e. lace monitor activity at carrion would be higher in summer and closed habitats), we constructed a generalised linear model (GLM) using the ‘MASS’ package (Venables & Ripley, 2002). The count of lace monitor events at each carcass site was used as the dependent variable whilst season and habitat were fitted as respective fixed effects alongside the interaction of both season and habitat as predictors. As data were over‐dispersed, we fitted a negative‐binomial link function to the model. To test the second part of prediction 1 (i.e. lace monitors occur at carcasses during earlier stages of carcass decomposition regardless of season and habitat), we constructed a linear mixed model (LMM) using the ‘lme4’ package (Bates et al., 2015). The LMM used ‘time since deployment’ (a measure of the number of days after carcass deployment that each independent event occurred) as the dependent variable. The predictor variables were season and habitat as fixed effects as well as the interaction between habitat and season, with carcass site applied as a random effect. Before running this model, we log transformed the data to satisfy the assumptions of the analyses.

For prediction 2 (i.e. lace monitors would locate carcasses slower than dingoes but at similar times to red foxes), we constructed a Cox proportional‐hazards model using the packages ‘survival’ (Thernau, 2020) and ‘timereg’ (Martinussen and Holst, 2023) that considered the time to arrival (time elapsed since carcass deployment and the first event) at each carcass site (in days) for lace monitors, dingoes and red foxes. This method was used because data were right censored, as not all carcasses were discovered by each species. We created a model with the response variable being time to arrival and with the predictor variables being the species type (lace monitors, red foxes and dingoes) and the interactions between species type and habitat (open and closed) and species type and season (winter and summer). Validating the model suggested violation of the proportional‐hazards assumption. Therefore, we modelled a multiplicative hazards model (Zhang et al., 2018). Hazard ratios were obtained from the model output. Finally, we computed Kaplan–Meier curves to compare arrival times visually. The proportional‐hazards assumption was assessed by plotting Schoenfield residuals against time.

For prediction 3 (i.e. There would be temporal separation in the use of carrion by lace monitors compared to dingoes and red foxes), we determined the time (24‐h time) that each event occurred for lace monitors, dingoes and foxes in summer and winter. We then compared the times that lace monitors were present with that of red foxes and dingoes within each season using a Watsons two‐sample test of homogeneity from the package ‘circular’ (Agostinelli & Lund, 2022). This technique is appropriate for comparing data that is bounded within a circular range, such as time data (Landler et al., 2021). From this, we calculated the circular mean for when the three species were active at carcass sites between seasons, bootstrapping 95% confidence intervals for the average time that each species was active at carcasses between both seasons.

To test prediction 4 (i.e. carcass sites with higher activity of dingoes and red foxes would have lower activity of lace monitors), we constructed a negative binomial GLM, with the count of lace monitor events at each site as a response variable and both dingo and red fox event counts as predictors. We were unable to include these variables as predictors in the GLM used to assess prediction 1 due to issues with model stability and convergence, thus we computed separate models to answer both predictions. All statistical analyses were conducted using R (Version 4.3.1) (R Core Team, 2019). Diagnostic plots were inspected to validate all GLMs and the LMM and we considered a significant effect where α < .05.

## 3 RESULTS

Lace monitors were active at 42 of the 80 carcasses deployed (52.5%). Carcass discovery by lace monitors was much higher in summer (*n* = 20 for closed sites and *n* = 18 for open sites [95% of total sites in summer]) than in winter (*n* = 3 for closed sites and *n* = 1 for open sites [10% of total sites in winter]). This resulted in 1076 independent events at carcasses, the species active both individually (*n* = 969 events) and occasionally in groups (*n* = 107 events), with a mean group size of 1.1 ± 0.01 (SE) (range: 1–4). Of the events that recorded more than one lace monitor, the average group size was 2.12 ± 0.04 (SE), including 96 events featuring two, nine events with three and two events with four individuals. These events contained adults only, with active scavenging typically dominated by the largest individuals present. However, instances occurred whereby multiple lizards fed simultaneously, though this typically ended at some point during the event with an aggressive interaction initiated by the largest individual present. Lace monitors appeared in 1056 events during summer and 20 in winter. When scavenging, lace monitors were observed (via cameras) consuming large amounts of the carcass biomass, even consuming bones and other recalcitrant matter typically difficult to ingest for many species that scavenge. All events in winter involved active scavenging on the carcass by the species and in summer 93.8% of events involved scavenging. Comparatively, dingoes occurred in a total of 430 events in summer and 403 in winter, feeding during 59% of summer events and 67% of winter events. Meanwhile, red foxes were active in 357 events in summer and 248 events in winter, feeding on carcasses in 52% of events during winter and 42% during summer.

### 3.1 Prediction 1: Lace monitor activity at carrion is higher in summer and closed habitats

**FIGURE 3:**
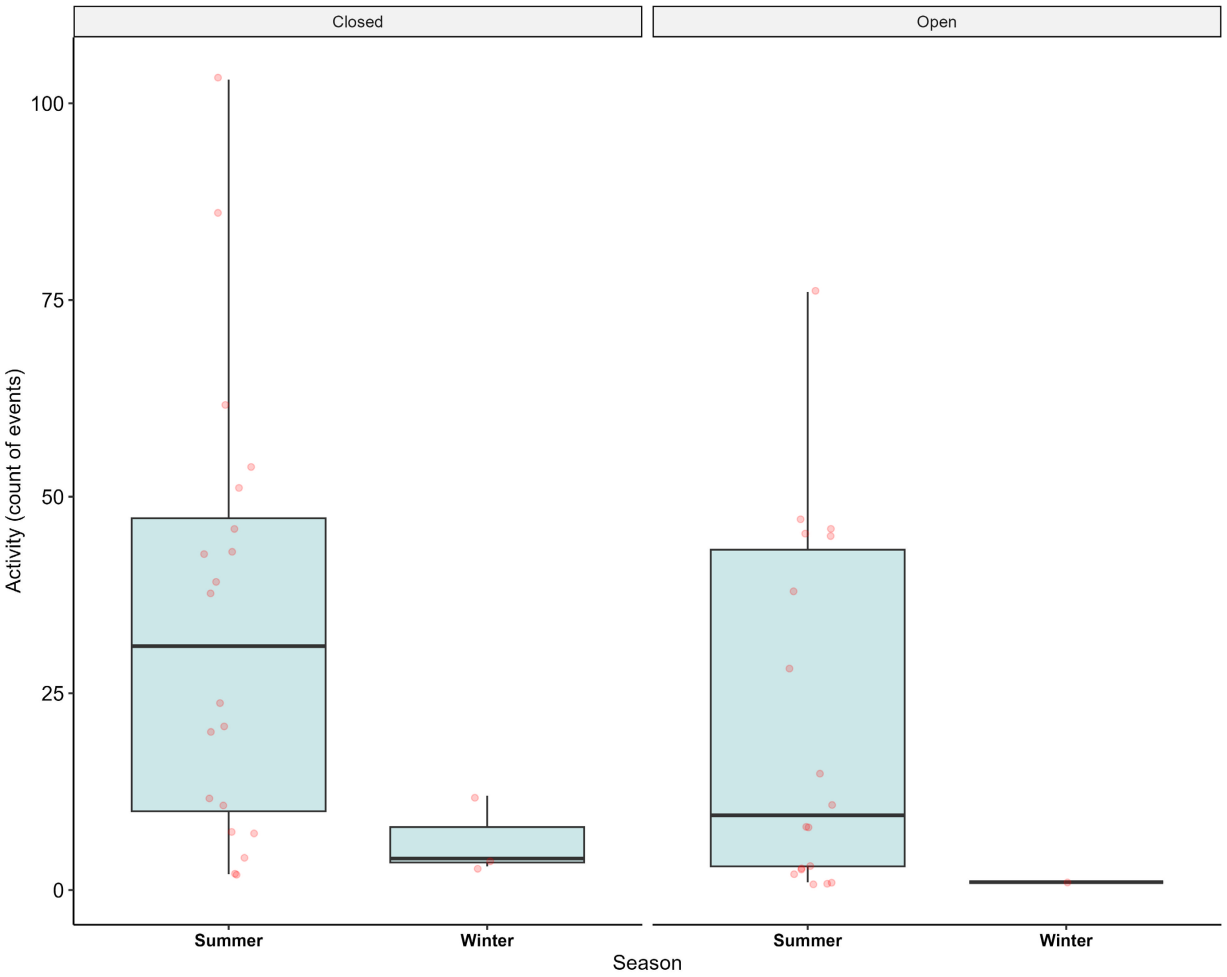
Boxplot showing total counts of lace monitor events at carcass sites between cool and warm seasons, stratified over open and closed habitats. Average lace monitor events (±SD) in summer open habitats: 21.17 ± 22.78 (*n* = 381) and closed habitats: 33.75 ± 28.25 (*n* = 675), in winter closed habitats: 6.33 ± 4.93 (*n* = 19). Note there was only a single event in open habitat during winter.

**FIGURE 4:**
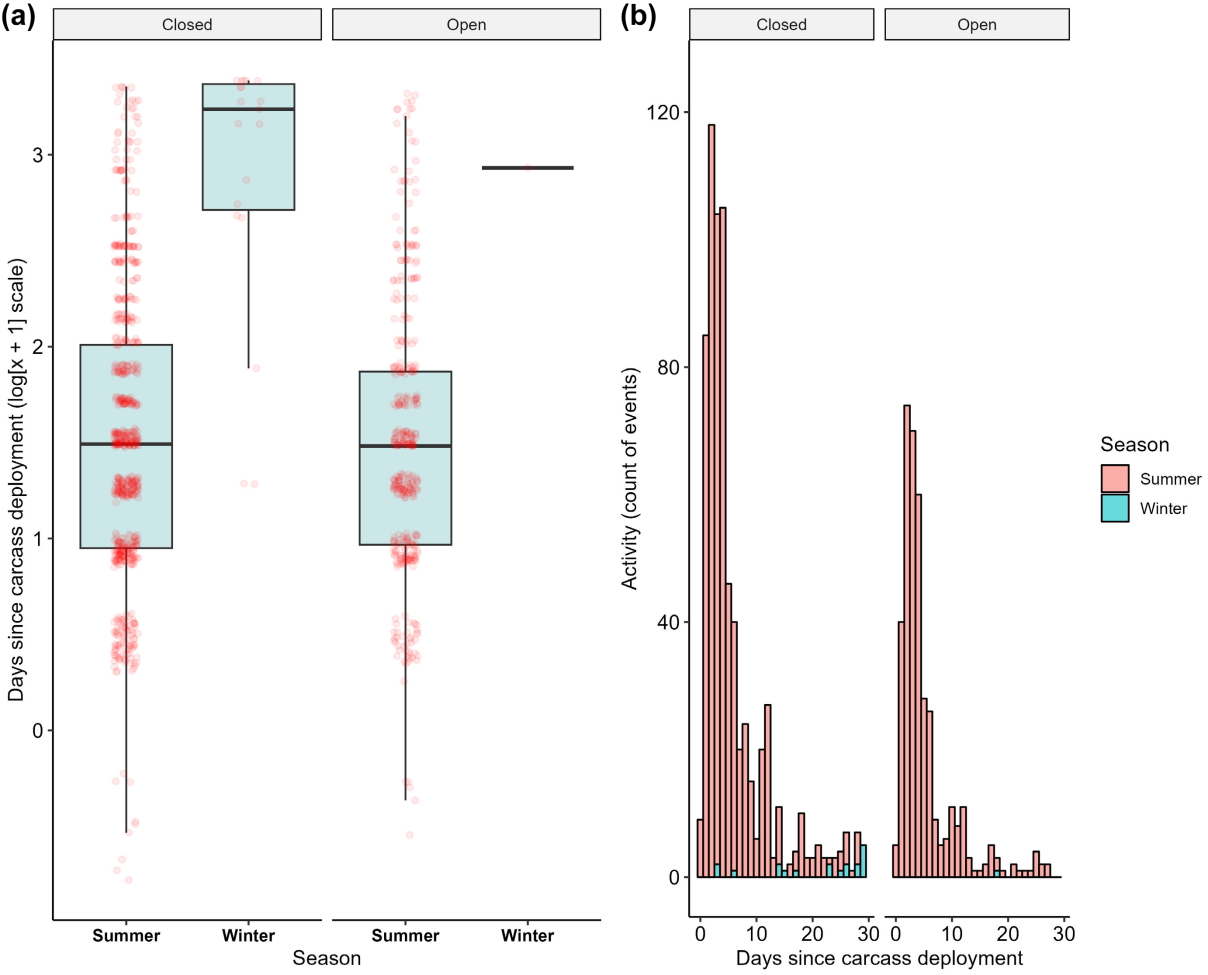
(a) A boxplot of the time each lace monitor event occurred after carcass site deployment, natural log transformed, across summer and winter and faceted between open and closed habitats. (b) A histogram of the number of lace monitor events across the maximum 30 days of monitoring carcasses in both summer and winter and facetted over open and closed habitats, binned into 1‐day intervals. Average time that events occurred (±SD) was 6.13 days ± 5.48 in summer closed habitats and 5.75 days ± 5.08 in open habitats. In winter closed habitats the mean was 21.40 days ± 9.15. The single event from winter in open habitats occurred 18.76 days after carcass deployment.

A significant effect was detected for lace monitor activity between seasons, with 1.67‐fold less events at carcasses in winter than summer (*Z* = −2.53, *p* = .01) (Figure 3). No significant effect was detected for habitat (*Z* = −1.41, *p* = .16), although less events occurred in open (*n* = 382 events, mean: 20.11 ± 1.16 [SE]) than closed habitats (*n* = 694 events, mean: 30.17 ± 1.06 [SE]). There was also no effect of the interaction between habitat and season on lace monitor activity at carcasses (*Z* = −0.80, *p* = .43). In summer, most events at carcasses for lace monitors occurred within the first 10 days of monitoring (mean: 5.99 ± 0.16 [SE] days), whereas events in winter occurred later in the monitoring period (mean: 21.26 ± 2.00 [SE] days) (Figure 4). Support for this was provided by the LMM, with lace monitor activity at carcasses occurring 1.47‐fold later (in log‐transformed days) in winter than summer (*t* = 8.28, *p* < .001). Additionally, lace monitor activity occurred slightly earlier across sites in open habitats (mean: 5.78 ± 0.26 [SE] days) compared to closed habitats (mean: 6.55 ± 0.23 [SE] days). Again, the model supported this statistically, with activity 0.22‐times earlier (in log‐transformed days) in open habitats than closed (*t* = −3.24, *p* = .001) (Figure 4). There was no effect for the interaction between season and habitat (*t* = 0.34, *p* = .74).

### 3.2 Prediction 2: Lace monitors locate carcasses slower than dingoes but at similar times to red foxes

**FIGURE 5:**
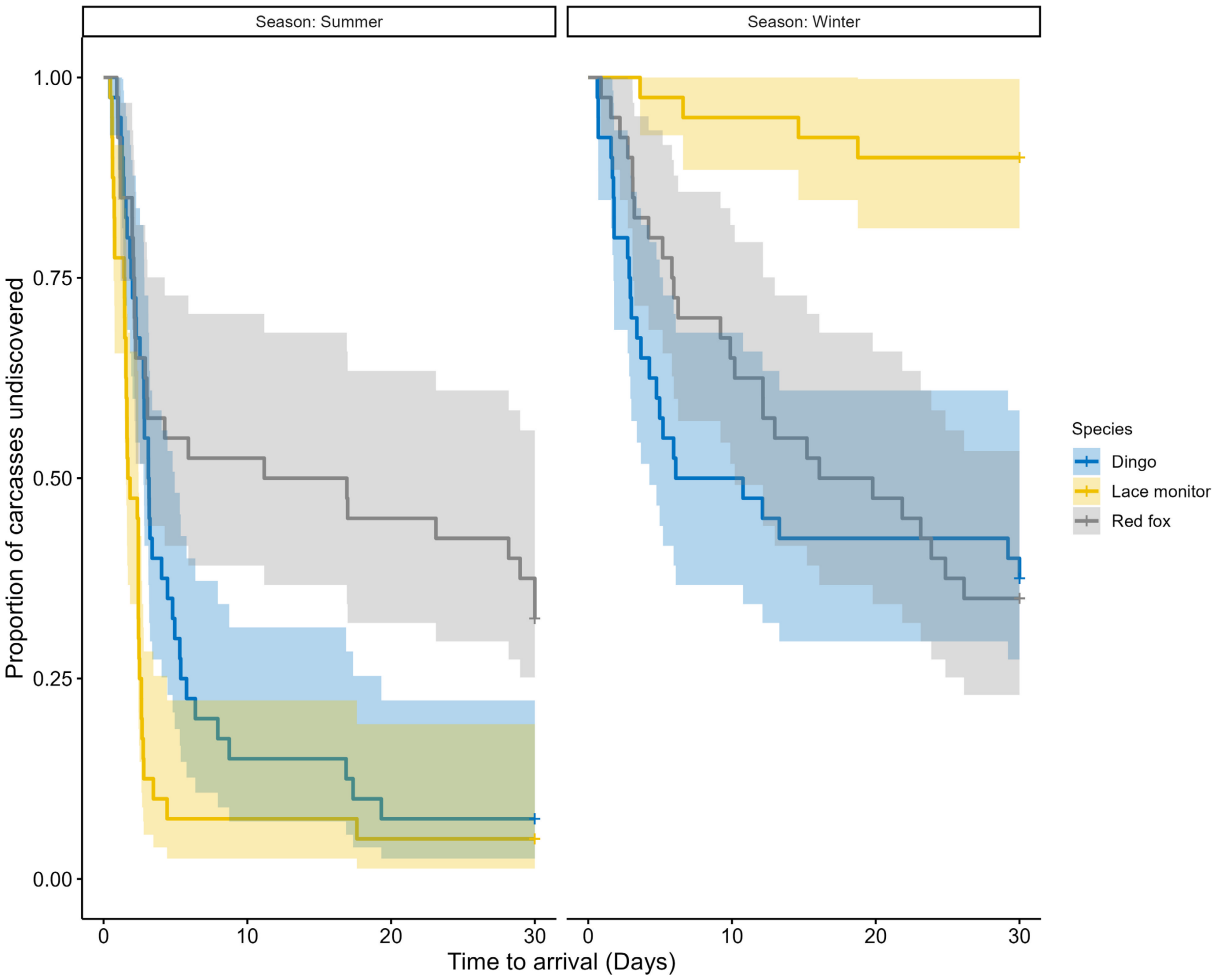
Kaplan–Meier survival curves depicting proportion of carcass sites remaining undiscovered by lace monitors, dingoes and red foxes across both winter and summer seasons for the 30 days spent monitoring. The shaded areas represent 95% confidence intervals.

A statistical effect was found for the interaction of lace monitors and season, with the species 3.95‐times more likely to discover carcasses during summer (Table 1 and Figure 5). Likewise, when comparing lace monitors to the reference level (dingoes) for time to first arrival at carcasses, lace monitors were 1.29‐times faster (in days) to find carcasses (Table 1 and Figure 5). In comparison, red foxes were 0.77‐times slower compared to the reference level (Table 1). No significant effect for the interaction between lace monitors with habitat was found and neither dingo nor red fox arrival times were influenced by habitat either (Table 1). Similarly, the discovery of carcass sites by red foxes was not influenced by season, however, for dingoes, initial arrival was 0.91‐times slower in winter than summer (Table 1 and Figure 5).

**TABLE 1:** Output of the multiplicative hazards models assessing time to arrival at carcasses for the three species across open and closed habitats and winter and summer seasons.

Variables	Coefficient	Hazard ratio	SE	*Z*‐value	*p*‐value	Hazard ratio – lower CI 97.5%	Hazard ratio – upper CI 97.5%
Species‐Lace monitor	1.290	3.633	0.321	2.790	**.005** *	1.917	6.753
Species‐Red fox	−0.765	0.465	0.326	−2.110	**.035** *	0.247	0.882
Species‐Dingo: Season‐Winter	−0.913	0.401	0.262	−2.810	**.005** *	0.239	0.671
Species‐Lace monitor: Season‐Winter	−3.950	0.019	0.553	−9.320	**<.001** *	0.007	0.057
Species‐Red fox: Season‐Winter	−0.190	0.827	0.282	−0.709	.478	0.476	1.438
Species‐Dingo: Habitat‐Open	0.317	1.373	0.259	1.010	.312	0.826	2.282
Species‐Lace monitor: Habitat‐Open	−0.809	0.445	0.321	−1.650	.099	0.239	0.839
Species‐Red fox: Habitat‐Open	0.208	1.231	0.282	0.774	.439	0.708	2.140

* Is assigned to variables that have a statistical effect *p* < .05.

### 3.3 Prediction 3: Lace monitors will use carrion at different times compared to dingoes and red foxes

**FIGURE 6:**
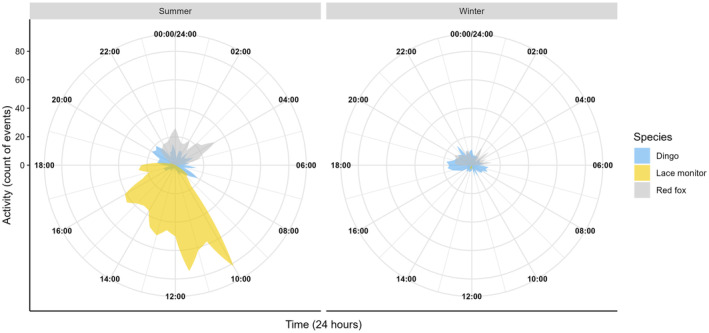
Rose plots showing activity times (in 24‐h time) for the lace monitor, dingo and red fox between winter and summer. In summer, the average activity time for lace monitors was 13:14 (95% CI: 13:04–13:24), whereas for the dingo it was 00:18 (95% CI: 22:49–01:54) and the red fox was 01:23 (95% CI: 01:04–01:41). In winter, the average activity time for lace monitors was 13:18 (95% CI: 12:42–13:58) whereas for dingoes it was 20:53 (95% CI: 19:28–22:30) and red foxes was 23:53 (95% CI: 23:15–00:32).

The temporal activity of lace monitors was significantly different for dingoes (*U* ^2^ = 16.52, *p* < .001) and red foxes (*U* ^2^ = 22.21, *p* < .001) in summer. Similarly, in winter, lace monitor activity differed between dingoes (*U* ^2^ = 1.20, *p* < .001) and red foxes (*U* ^2^ = 1.50, *p* < .001). When plotted, it was evident that lace monitors were strictly diurnal whilst red foxes were nocturnal (Figure 6). Dingoes displayed some diurnal activity, but this was much less significant than their nocturnal activity (Figure 6).

### 3.4 Prediction 4: Carcasses with higher dingo and red fox activity have lower activity of lace monitors

**FIGURE 7:**
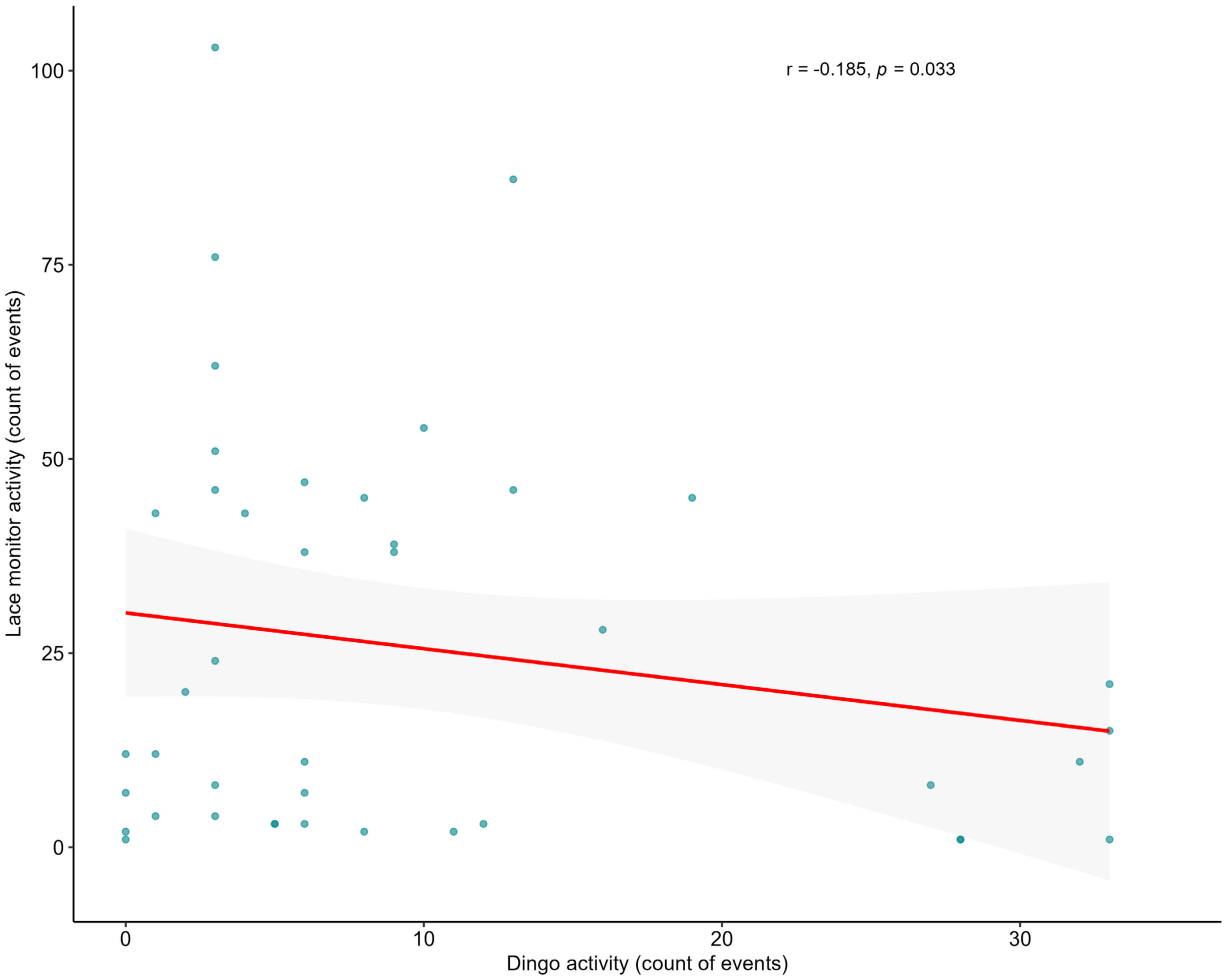
Scatterplot depicting the number of events for both lace monitors and dingoes when both species co‐occurred at carcass sites. The Pearson correlation coefficient for the data (*r*) is −.185 and is shown on the plot. The trendline for the GLM regression is in red, with standard error shaded.

Increasing red fox activity at carcass sites did not correlate to less lace monitor activity at carcass sites (Table 2). Contrarily, for dingoes, we found a statistical effect, with increasing dingo activity at carcass sites correlated with a slight 0.04‐fold decrease in activity for lace monitors (Table 2 and Figure 7).

**TABLE 2:** Output of the negative binomial GLM assessing lace monitor activity at carcass sites against dingo and red fox activity.

Variables	Estimate	SE	*Z*‐value	*p*‐value
Intercept	3.664	0.266	13.801	**<.001** *
Dingo	−0.036	0.017	−2.137	**.033** *
Red fox	−0.016	0.012	−1.333	.183

* Is assigned to variables that have a statistical effect *p* < .05.

## 4 DISCUSSION

Here we have quantified the foraging activity of lace monitors at medium‐sized (~30 kg) carcasses and have explored potential biotic and abiotic factors that influence their use of these nutrient‐dense resources. Current knowledge of this species underscores the importance of animal carcasses within their diet, alike to other varanids (Guarino, 2001; Pettit, Ward‐Fear, & Shine, 2021; Weavers, 2014), but no study to date has explored their scavenging behaviours in detail. Because the species is able to shift foraging patterns based on resource availability alongside other environmental conditions (Jolly et al., 2016; Pettit et al., 2020), lace monitors are likely effective exploiters of carrion. Our study supported this notion, as we found that lace monitors featured prominently at carcass sites. Indeed, lace monitors occurred at over 50% of carcasses studied, with over 90% of carcasses visited in warmer summer months. We found that rates of lace monitor activity were mediated strongly by season. As predicted (prediction 1), lace monitors were exponentially more active in summer than winter months. On the other hand, against prediction 1, the species activity was not found to differ significantly at carcasses in closed and open habitats, across both summer and winter. Also, against prediction 1, lace monitors were active at carrion marginally earlier in open compared to closed habitats, and they occurred at much later times at carcasses in winter than in summer months. Against our prediction 2, in summer, lace monitors were faster to locate carcasses than both dingoes and red foxes, across all habitats. Finally, despite lace monitors active at significantly different times to dingoes and red foxes over both summer and winter (in support of our prediction 3), we found that lace monitor activity declined with higher levels of dingo but not red fox activity (mixed support for prediction 4).

### 4.1 Season and habitat effects on lace monitor scavenging

It was unsurprising that we found lace monitors to be far more active at carcasses in summer than winter months, as this species is known to limit its daily activity at lower temperatures and cooler seasons (Pascoe et al., 2019; Stebbins & Barwick, 1968). In summer, carcass use by monitors also generally occurred earlier, within the first 10 days after deployment. This corresponds to the period when there is the most edible biomass available on a carcass (Moleón et al., 2019), as invertebrate scavengers can rapidly break down carrion in these warmer months (Barton & Bump, 2019). Additionally, the odour cues used to find carrion may be lessened in winter, with the volatile organic compounds emitted by carrion, which animals use to locate the resource, typically produced less in winter than warmer periods (Forbes et al., 2014). Contrastingly, lace monitors exhibited similar activity across carcass sites in closed and open habitats, which may have related to the proximity of carcasses in open habitats to forested habitats, and the relatively mobile nature of large varanids (Pascoe et al., 2019; Thompson, 1992). While this species typically prefer forested areas (Lei & Booth, 2018b), carcasses in open habitats might be easier to detect, as they are less obscured by vegetation (Krige et al., 2024; Pardo‐Barquín et al., 2019). This could also partially explain why lace monitors were active earlier at carcasses in open compared to closed habitat (i.e. more individuals were able to more rapidly locate carcasses in open habitats), but this result may also potentially be related to the conditions that carcasses were exposed to. In open environments, for example, carcasses are more exposed to abiotic elements such as wind, precipitation and solar irradiation, which denude biomass quicker than those sheltered by vegetation (Moleón et al., 2019). As a result, there may be advantages to earlier activity at carcass sites in open habitats, such as access to higher quality resources. Thus, meaning there was less pressure for lace monitors to immediately feed within closed habitats where biomass may be preserved for longer. There is also the likelihood that individual traits influenced this finding, with bolder or larger lizards moving to carcasses in open habitats to monopolise on a resource that may have less competition from conspecifics, since boldness in individual varanids is an important part of their biology and influences habitat selection, movement patterns and foraging among other factors (Pettit, Brown, et al., 2021; Ward‐Fear et al., 2018).

### 4.2 Lace monitor, dingo and red fox scavenging

Lace monitors use a combination of olfactory and visual cues to locate food (Cooper Jr., 1989; Garrett et al., 1996; Garrett & Card, 1993; Kaufman et al., 1996). These attributes help them to forage generally, and to find carcasses and exploit this resource where it occurs. However, dingoes and red foxes also have good sensory adaptations that enable them to forage effectively (Jacobs et al., 1993; Ortiz‐Leal et al., 2022). Furthermore, as larger, more mobile species, dingoes and red foxes could be expected to have a competitive advantage in terms of carcass discovery over smaller, comparatively less mobile species, like lace monitors. Despite this, we found that lace monitors discovered carcasses quicker compared to red foxes and dingoes. This finding could be explained by differences in dietary preferences influencing decisions to visit and scavenge carcasses. Specifically, the proportion of carrion in the diets of both dingoes (Corbett & Newsome, 1987; Thomson, 1992) and red foxes (Lacombe et al., 2024; O'Connor et al., 2020; Sivy et al., 2018) likely varies depending on background factors such as prey availability. In contrast, it is thought that lace monitors are willing to scavenge regardless, eating whatever food items are easiest to access (Guarino, 2001). It is also probable that densities of these scavenging species in the surrounding environment influenced rates of carcass detections. That is, if a species was highly abundant in the area surrounding the carcass, there would be a higher likelihood of them encountering that carcass. Monitoring baseline densities of lace monitors and other scavengers like the red fox and dingo prior to carcass placement would be useful in future studies.

That lace monitor activity at carrion did not change in relation to the use of the resource by red foxes is probably due to several reasons. Foremost, we found that lace monitor and red fox activity at carrion did not overlap temporally, with red foxes strictly nocturnal and lace monitors diurnal, in line with other research (Ables, 1969; Moore et al., 2020; Weavers, 1993). Such temporal partitioning would have reduced possible inter‐specific conflict. Furthermore, whilst being a prolific scavenger when active at carcass sites, the red fox may not have been able to remove large enough amounts of biomass to compete with lace monitors. Indeed, we found that whilst red foxes were positively correlated with the removal of carcass biomass within our study this relationship was not significant in comparison to that of the dingo (Appendix S1).

On the other hand, dingoes, as apex scavengers, can effectively remove large amounts of carrion when scavenging and dominate the scavenger community (Newsome et al., 2024). It was therefore not surprising that, when dingoes had higher levels of activity at carcass sites, we found a corresponding decrease in the activity of lace monitors. This was despite the temporal differences in carcass use between dingoes and lace monitors, although there was more overlap in activity times in summer between dingoes and lace monitors than between red foxes and lace monitors. Dingoes may prey on lace monitors (Webb, 1996), so there is the potential that lace monitors avoided carcass sites with higher dingo activity, as has been observed in previous studies between lace monitor and other predators (Anson et al., 2014; Hu et al., 2019). However, although some diurnal activity was recorded for dingoes, we found no instances of direct interaction between the two species. Additionally, the levels of dingo activity that occurred diurnally were vastly lower than at night, when lace monitors were not active. It is therefore most likely that high dingo activity, and corresponding scavenging at carcass sites, resulted in less carcass biomass available for lace monitors, reducing their activity at these sites. This is a typical relationship observed in scavenger communities (Beasley et al., 2015; O'Bryan et al., 2019) and provides an example of how a larger predator species may outcompete lace monitors. Nonetheless, lace monitors were effective scavengers and may be important removers of carcasses in landscapes without apex scavengers, such as degraded agricultural areas where dingoes have been historically controlled. Indeed, this ecological role has been suggested for a similar but smaller monitor species (Jameson et al., 2024). With the commonness of carrion in the diet of lace monitors (Guarino, 2001; Pascoe et al., 2011), and their larger distribution across Australia (Smissen et al., 2013), we speculate it is likely lace monitors may play an even more important role in this function than other species of monitor.

### 4.3 Limitations, implications and future directions

Our methodology would have been improved by capturing the activity and diet (e.g. through unbaited camera trap surveys and scat or stomach analysis) of lace monitors, alongside dingoes and red foxes, prior to beginning our carcass deployments. This would have allowed for more detailed conclusions to be drawn about not only the activity of this varanid species and its interactions with these two mammalian predators, but the relative importance of carrion in its diet. Additionally, we did not ascertain the relative abundance of non‐carcass food items in the surrounding environment for lace monitors, or for red foxes and dingoes, prior to and during each carcass deployment period. The availability of alternative food items may dictate the activity of species at carcasses, particularly for the red fox and dingo (Corbett & Newsome, 1987; Glen et al., 2007; Paltridge, 2002; Pascoe et al., 2011; Weavers, 2014). Thus, understanding how shifts in food availability between seasons and carcass deployment periods interact with the activity of lace monitors and other competing scavengers at carcass sites would be extremely useful. For example, monitoring could be undertaken in a region before and after management programmes that cull kangaroos or other herbivores to determine how a sudden increase in carrion biomass on a landscape‐scale influences lace monitor diets and resulting interactions with competing meso and apex predators.

We suggest that further scavenging research should also focus on surveying the activity and foraging behaviours of other reptile species that potentially scavenge. Monitoring reptiles is often difficult due to their biology and cryptic nature, requiring intensive survey efforts. Further, reptiles have generally been overlooked in scavenging studies, alongside other aspects of ecological research, more broadly (Guedes et al., 2023). Adopting a systematic carrion‐baited camera trap survey design may yield higher detection rates than other methods. Camera trapping surveys for larger reptiles are likely to be a more effective way of monitoring these animals and has been used before with success (Moore et al., 2020). Furthermore, the use of a bait or carcass provides a means of increasing the chance that cryptic or rare reptile species are detected (Pettit, Ward‐Fear, & Shine, 2021). Other studies have successfully used meat baits and small carcasses to attract difficult‐to‐detect species, for example, to monitor lace monitors and threatened yellow‐spotted monitors (*Varanus panoptes*) in northern Australia (Pettit, Ward‐Fear, & Shine, 2021). With active search‐based sightings of yellow‐spotted monitors known to be difficult with variable rates of detection and reliability (Ward‐Fear et al., 2019), this method proved to be a viable alternative. There is a potential utility for this to be extended to other threatened monitors such as the heath monitor (*Varanus rosenbergi*) in eastern Australia and the Komodo dragon (*Varanus komodoensis*) in Indonesia, varying carcass size based upon the biology and dietary preferences of the species targeted (e.g. Ariefiandy et al., 2014). Finally, based on our findings, we also propose that such a method is best used in periods of higher reptile activity (e.g. in summer), but should also be applied across different habitats (e.g. closed forests and open grasslands). Nonetheless, researchers must consider factors like co‐occurring scavenger species (especially apex scavengers like the dingo) as this might influence reptile occurrence at carcass monitoring sites.

## AUTHOR CONTRIBUTIONS

**Rhys J. Cairncross:** Conceptualization (lead); formal analysis (lead); writing – original draft (lead). **Emma E. Spencer:** Data curation (lead); writing – review and editing (equal). **Niraj Meisuria:** Conceptualization (supporting); writing – review and editing (supporting). **Mathew S. Crowther:** Formal analysis (supporting). **Thomas M. Newsome:** Conceptualization (supporting); writing – review and editing (equal).

## CONFLICT OF INTEREST STATEMENT

The authors declare no conflicts of interest.

## Supporting information


Appendix S1.

## Data Availability

Data are available online at the following repository: https://doi.org/10.5061/dryad.0p2ngf28m.
