# Supplementary material for: Carrion use by a reptile is influenced by season, habitat and competition with an apex mammalian scavenger
Source: Ecol Evol. 2024 Aug 28;14(8):e70211. doi: 10.1002/ece3.70211 (PMC11350275; doi:10.1002/ece3.70211)
Supplement: Supplementary file 1 — Appendix S1. [file ECE3-14-e70211-s001.docx]

Appendix A

Context:

To quantify the contribution of both dingoes (*Canis dingo*) and red foxes (*Vulpes vulpes*) to carcass biomass loss, we determined the number of scavenging events that both species contributed to in the first 14 days of monitoring at a subset of carcass sites from our dataset (n = 40 total, n = 20 across both summer and winter seasons). This timeframe was used as, typically, the most biomass removal occurs in this period (Spencer and Newsome 2021). We then totalled the number of events for both species and ran a generalised additive model with gaussian distribution predicting the amount of carcass biomass removed (in kilograms) that both contributed to. We fitted both dingo and red fox scavenging events as parametric (fixed) effects and as smoothed splines to account for potential non-linear relationships in the data. The removal of carcass biomass by both species may result in reduced feeding opportunities for other scavengers, thereby disincentivising activity for species like lace monitors (*Varanus varius*) that attend carcasses.

Results:

The model found a statistical effect, whereby dingoes contributed to a 1.098-fold increase in carcass biomass removal (t = 5.72, p < 0.001). Higher activity of red foxes was also correlated with an increase in carcass biomass removal (0.21-fold increase), but this did not exhibit a statistical effect (t = 1.17, p = 0.25). The smooth for dingo activity also received statistical support (F = 8.19, p < 0.001), with rates of biomass removal increasing exponentially before exhibiting some variability at higher levels of dingo activity (Figure A1). However, for red foxes, we did not find a statistical effect for the smoothed term (F = 18.16, p = 0.24).

Discussion:

The results indicate that both dingoes and red foxes contribute to increased rates of carrion biomass removal, but that dingoes are likely to have a stronger influence on this. Nonetheless, higher rates of activity at carcass sites for both species together is likely to result in other scavenger species potentially having fewer foraging opportunities. This competition is not uncommon in scavenger communities, especially when apex scavengers like dingoes dominate (Wikenros *et al.* 2013; Jung *et al.* 2023).

References:

Jung TS, Peers MJL, Drummond R, Taylor SD (2023). Dining with a glutton: an intraguild interaction between scavenging wolverine (Gulo gulo) and lynx (Lynx canadensis). *Ecosphere* **14**, e4491. doi:10.1002/ecs2.4491

Spencer E, Newsome T (2021). Dingoes dining with death. *Australian Zoologist* **41**, 433–451. doi:10.7882/AZ.2021.008

Wikenros C, Sand H, Ahlqvist P, Liberg O (2013). Biomass Flow and Scavengers Use of Carcasses after Re-Colonization of an Apex Predator. *PLOS ONE* **8**, e77373. doi:10.1371/journal.pone.0077373

Figures:


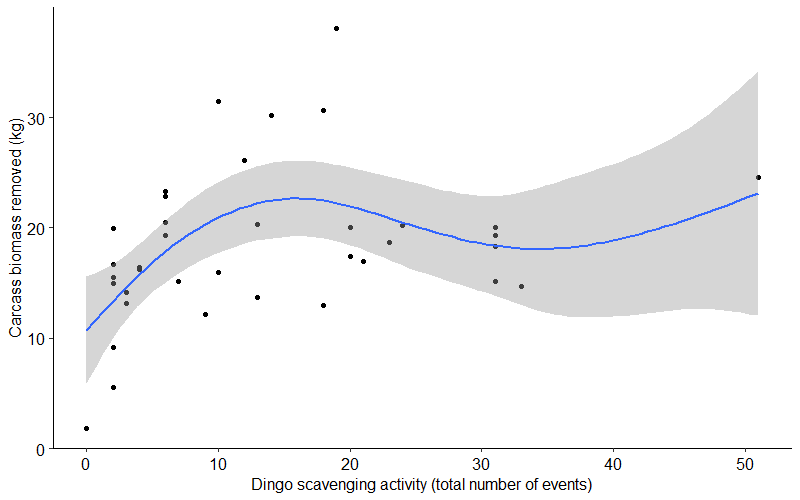


Figure A1: Scatterplot with smoothed term demonstrating the relationship between dingo scavenging activity and the amount of carcass biomass removed.
